# Enhancement of intestinal mucosal immunity and immune response to the foot-and-mouth disease vaccine by oral administration of danggui buxue decoction

**DOI:** 10.3389/fvets.2022.1045152

**Published:** 2022-11-08

**Authors:** Bingxin Zhou, Huan Huang, Fuxing Gui, Shicheng Bi, Hongxu Du, Liting Cao

**Affiliations:** ^1^Department of Traditional Chinese Veterinary Medicine, College of Veterinary Medicine, Southwest University, Chongqing, China; ^2^Immunology Research Center, Medical Research Institute, Southwest University, Rongchang, Chongqing, China; ^3^Chongqing Engineering Research Center of Veterinary Medicine, Chongqing, China; ^4^Chi Institute of Traditional Chinese Veterinary Medicine, Southwest University, Chongqing, China

**Keywords:** Danggui Buxue decoction, foot-and-mouth disease vaccine, humoral immunity, intestinal mucosal immunity, immune enhancement

## Abstract

This study investigated the effect of Danggui Buxue decoction (DBD) on the immunity of an O-type foot-and-mouth disease (FMD) vaccine and intestinal mucosal immunity. SPF KM mice were continuously and orally administered DBD for 5 d and then inoculated with an O-type FMD vaccine. The contents of a specific IgG antibody and its isotypes IgG1, IgG2a, IgG2b, and IgG3 in serum and SIgA in duodenal mucosa were determined by ELISA at 1 and 3 W after the 2^nd^ immunization. qRT-PCR was used to detect mRNA expression levels of IL-4, IL-10, IFN-γ, and IL-33 in the spleen, and mRNA expression levels of J-chain, pIgR, BAFF, APRIL, IL-10, IFN-γ and IL-33 in the duodenum. The results showed that compared with the control group, oral administration of DBD significantly increased levels of the anti-FMD virus (FMDV)-specific antibodies IgG, IgG1, and IgG2a in the serum of O-type FMD vaccine-immunized mice 1 W after the 2^nd^ immunization (*P* < *0.05*), upregulated mRNA expression levels of spleen lymphocyte cytokines IL-4 and IL-33 (*P* < *0.05*), promoted the secretion of SIgA in duodenal mucosa (*P* < *0.05*). The mRNA expression levels of J-chain, pIgR, BAFF, APRIL, IL-10, and IL-33 in duodenal tissues were upregulated (*P* < *0.05*). This study indicates that DBD has a good promotion effect on the O-type FMD vaccine and the potential to be an oral immune booster.

## Introduction

Foot-and-mouth disease (FMD) is a highly contagious acute infectious disease caused by FMD virus (FMDV) and mainly affects cloven-hoofed animals such as pigs, cattle, goats, and camels (1). The incidence of FMD is extremely high and the transmission speed is extremely fast. FMDV is mainly transmitted through the digestive tract, respiratory tract, skin, and mucosa. Mucosal damage in the early stage of infection is highly susceptible to infection by other bacteria in the external environment (2). In today's intensive farming environment, once an individual gets sick, a group infection will soon break out and cause serious economic losses to the breeding industry (3). Presently, vaccination is the most important means for the prevention and control of FMD (4). Therefore, compulsory immunization with FMD vaccine was adopted in China to prevent and control the disease, which can control a possible pandemic of FMD to a certain extent (5). However, due to the uneven level of breeding health management in some areas, the phenomenon of poor immune effects of FMD vaccines has been reported from time to time (6). Therefore, it is necessary to develop an immune booster to improve the immune effect of the FMD vaccine.

Chinese herbal medicine comes from nature and has low toxicity and side effects. It also has a unique effect in coordinating the overall balance of the body and strengthening the disease-resistance ability. Recently, researchers have used modern science and technology to study traditional Chinese medicine and found that Chinese herbal medicine or effective active ingredients have significant immunomodulatory effects (7, 8). Danggui Buxue decoction (DBD) was created by Li Dongyuan, one of the four great scholars of the *Jin* and *Yuan* Dynasties. It is composed of *Radix Astragali* and *Angelica sinensis* and replenishes *Qi* and promotes the production of blood (9). Modern studies have confirmed that DBD can promote angiogenesis (10), improve bone marrow hematopoietic function (11), enhance non-specific immunity (12) and improve immune regulation in a pathological injury model (13). The intestinal tract is not only a place for digestion and absorption of nutrients but also has important immune functions. The intestinal mucosa is a key line of defense for the body to resist pathogen infection, and various defense mechanisms are conducted in the intestine, which play an important role in the establishment and maintenance of homeostasis between the host and the external environment (14). Chinese herbal medicine is a group of natural substances, which has the advantages of high safety, enhancing humoral and cellular immunity, non-toxic side effects, wide range of medication, etc. And it has been reported that a large number of herbal medicines or their extracts have been used as vaccine adjuvants, such as *Angelica sinensis* polysaccharide (15), ginsenoside Rg1 (16) and Qi-Wu Rheumatism Granule (17). Most Chinese herbal medicines are taken orally to exert effects and have a close relationship with the intestinal mucosal immune system. Therefore, this study was conducted to investigate the effect of oral DBD on the immune function of an O-type FMD vaccine and intestinal mucosal immunity in mice to provide a theoretical basis for the clinical application of traditional Chinese medicine.

## Materials and methods

### Reagents

*Angelica sinensis* and *Radix astragali* were purchased from Chongqing Rongchang Tongjunge pharmacy.

The O-type foot-and-mouth disease synthetic peptide vaccine (Peptide 2600+2700+2800) (20190408) was purchased from Shenlian Biomedicine (Shanghai, China) Co., Ltd. A mouse (FMDV-OIgG) ELISA kit (20190512), mouse FMDV-OIgG1, FMDV-OIgG2a, FMDV-OIgG2b, FMDV-OIgG3) ELISA kit (20190422), and mouse SIgA ELISA kit (20190612) were purchased from Shanghai Huzhen Industrial Co., Ltd. TRIzon Reagent (20190323), HiFi Script gDNA Removal RT Master Mix (30412), and 2xEs Taq Master Mix (50428) were purchased from Beijing Kangwei Century Co., Ltd. Eva GREEN (17E0.1-1075001) was purchased from Biotium, USA.

### Preparation of danggui buxue decoction

One hundred grams of *Radix astragali* and 20 grams of *Angelica sinensis* were accurately weighed, soaked in 8 times distilled water for 30 min with high heat until boiling, and then simmered gently for 1 h. The liquid was filtered and the residue was added to the same amount of distilled water for an additional 1 h. The filtrate was filtered again and combined. The final concentration of liquid was equivalent to 2 g/m L of the original medicine. The medicinal liquid was separated into 2, 1, 0.5, 0.25, 0.125, and 0.0625 g/mL DBD and stored at 4°C for later use.

### Animals

A total of 94 SPF KM mice weighing 20 ± 2 g were purchased from Chongqing Ensiweier Biotechnology Co., Ltd. All experimental mice were raised in an environment with a temperature of 20–25°C and a relative humidity of 60–80% and were freely allowed to feed and drink. After 1 W of adaptive feeding, the formal experiment began. This study was approved by the Laboratory Animal Ethical Review Committee of Southwest University (permit number IACUC-20211020-01), and all experimental animals were euthanized at the end of the study.

### Experimental design

**Figure 1:**
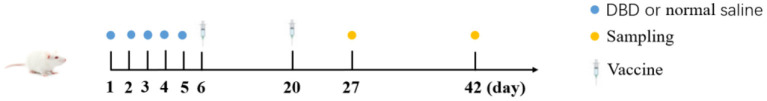
Grouping and processing in mice.

In experiment 1, 70 KM mice, half male and half female, were randomly divided into 7 groups (*n* = 10), which were 2, 1, 0.5, 0.25, 0.125 and 0.0625 g/mL DBD dose groups and control group. The dose was 0.25 mL/each, which was given by gavage for 5 d. The control group was given the same amount of normal saline. Twenty-four hours after the last dose, all experimental mice were subcutaneously injected with 0.2 mL of O-type FMD vaccine in the groin followed by a second immunization at 2 W later. The mice were weighed before immunization and 1 and 3 W after immunization, and blood samples were collected under the jaw at 1 and 3 W after immunization. All mice were sacrificed by neck removal at 3 W after immunization, and the spleen and thymus were aseptically harvested (Figure 1).

In experiment 2, 24 KM mice, half male and half female, were randomly divided into two groups (*n* = 12), which were the optimal dose group of DBD and the control group, respectively. The mice were treated according to the method of Experiment 1. All experimental mice were sacrificed by neck stripping 3 W after the 2nd immunization, and the spleen, duodenum, and mucosal flushing fluid were aseptically extracted (Figure 1).

### Determination of the immune organ index

The spleen and thymus of all experimental mice were accurately harvested, and the blood stains on the surface were washed with sterilized saline. Then, excess water on the organ surface was aspirated with clean filter paper and weighed.

Calculation formula: Organ index = organ weight (mg)/mouse weight (g).

### Detection of serum-specific antibody and duodenal SIgA

The serum and duodenal mucosa supernatant were obtained by separating the collected blood and duodenal mucosa rinse solution at 3,000 rpm/min for 10 min. The content of FMDV-specific antibody IgG in serum at 1 W and 3 W after the 2^nd^ immunization, specific antibody isotypes IgG1, IgG2a, IgG2b, and IgG3 in serum, and SIgA in duodenal mucosal rinse solution 3 W after the 2^nd^ immunization were detected according to ELISA kit instructions.

### RNA extraction and qRT-PCR

Total RNA was extracted from the spleen and duodenum using TRIzol Reagent and reverse transcribed into cDNA using HiFi Script gDNA Removal RT Master Mix and 2xEs Taq Master Mix. The mRNA expression levels of IL-4, IL-10, IFN-γ, and IL-33 in the spleen and APRIL, BAFF, pIgR, J-chain, IL-10, and IL-33 in the duodenum were detected by real-time PCR using Eva GREEN (Biotium17E0.1-1075001) with mouse β-actin as the internal reference. The results are expressed as 2^−ΔΔct^, and the primer sequences are shown in Table 1.

**Table 1:** Sequences of primers for qRT-PCR.

**Gene**	**Primer sequences (5^′^-3^′^)**	**bp**
*IL-4*	• F: CACAGCAACGAAGAACACCAC • R: TCTGCAGCTCCATGAGAACAC	131
*IL-10*	• F: GTTGCCAAGCCTTATCGGA • R: GCCGCATCCTGAGGGTC	132
*IL-33*	• F: GTCTCCTGCCTCCCTGAGTACATAC • R: AGTAGCGTAGTAGCACCTGGTCTTG	128
*IFN-γ*	• F: TTGCCAAGTTTGAGGTCAACAA • R: CGAATCAGCAGCGACTCCT	126
*APRIL*	• F: GTTAACATTACCTCCAAGGACTCT • R: TTCCAGTGTCCCAGACTCGTA	115
*BAFF*	• F: GTTGCCAAGCCTTATCGGA • R: GCCGCATCCTGAGGGTC	114
*pIgR*	• F: GGGTTGCCACATCCTGCCAAG • R: CACACCAGTACCAGCCTTCATCTTC	131
*J-chain*	• F: CTCTGATCCCACCTCCCCACTG • R: TCAGGAACACCATCGTCTTCATTGC	150
*β-actin*	• F: TATGCTCTCCCTCACGCCATCC • R: GTCACGCACGATTTCCCTCTCAG	129

### Statistics analysis

Data are expressed as the mean ± standard error (SE) and were analyzed by one-way ANOVA *post-hoc* Duncan's method and *t*-test with IBM SPSS Statistics 22.0 software. Differences with *P* < *0.05* were considered statistically significant.

## Results

### Body weight and immune organ index

As depicted in Tables 2, 3, the body weight (Table 2), spleen index, and thymus index (Table 3) of the experimental mice were not significantly affected by different doses of DBD compared with the control group (*P* > *0.05*).

**Table 2:** Effect of oral administration of DBD on the mean body weight in mice (*n* = 10).

**Group**	**Pre-immunization**	**2 W after 1st immunization**	**2 W after 2nd immunization**	**3 W after 2nd immunization**
0.0625 g/ml	20.3 ± 0.58	28.1 ± 1.09	34.7 ± 0.86	43.0 ± 0.63
0.125 g/ml	20.1 ± 0.53	28.3 ± 1.24	33.8 ± 0.51	42.7 ± 1.28
0.25 g/ml	20.1 ± 0.55	29.0 ± 0.96	34.2 ± 0.95	42.3 ± 0.94
0.5 g/ml	19.4 ± 0.48	29.0 ± 1.14	33.7 ± 0.52	42.1 ± 0.86
1 g/ml	20.4 ± 0.34	29.3 ± 1.04	33.3 ± 0.65	42.6 ± 1.31
2 g/ml	20.1 ± 0.41	28.8 ± 0.81	33.2 ± 0.61	43.8 ± 0.77
control	19.2 ± 0.55	29.7 ± 0.73	33.9 ± 0.82	43.3 ± 0.99

**Table 3:** Effect of DBD on the organ index in mice (*n* = 10).

**Group**	**Spleen index**	**Thymus index**
0.0625 g/ml	2.25 ± 0.17	4.48 ± 0.30
0.125 g/ml	2.50 ± 0.17	4.37 ± 0.32
0.25 g/ml	2.57 ± 0.11	4.64 ± 0.15
0.5 g/ml	2.97 ± 0.16	5.48 ± 0.23
1 g/ml	2.42 ± 0.13	5.27 ± 0.19
2 g/ml	2.70 ± 0.19	5.78 ± 0.26
control	2.67 ± 0.16	5.37 ± 0.17

### Serum IgG and its isotypes

**Figure 2:**
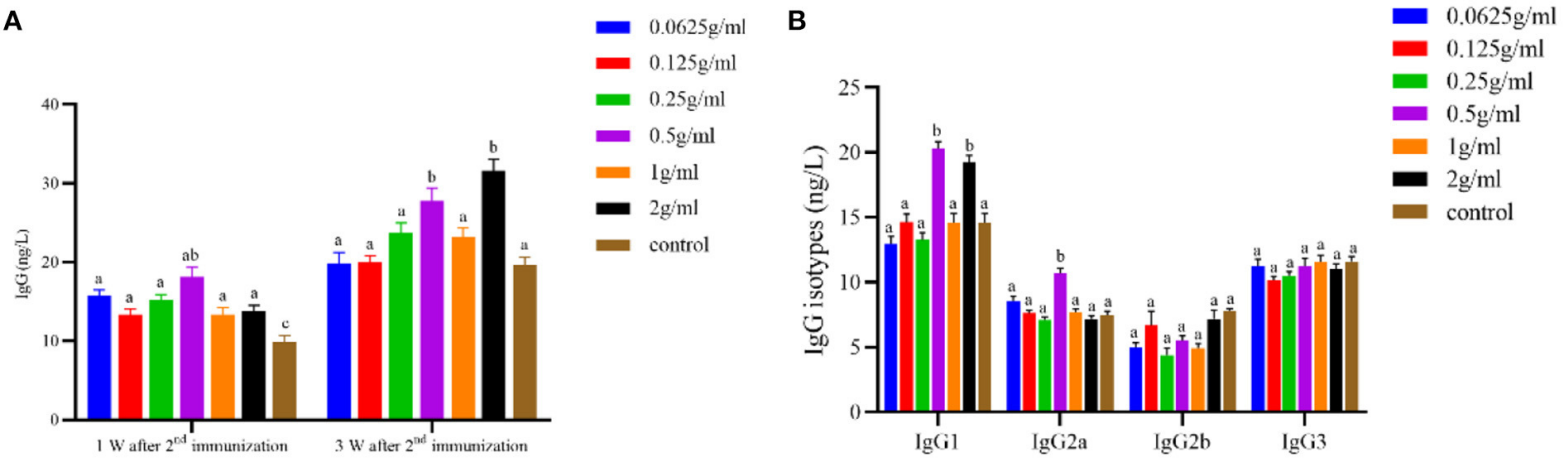
Effects of orally administered DBD on serum FMDV-specific antibody content in mice. **(A)** The concentration of IgG antibody. **(B)** The concentration of IgG isotypes antibody. ^a,b^Values in the same column with different superscript letters differ significantly at *P* < *0.05*.

As shown in Figure 2, the serum anti-FMDV-specific antibody IgG content in each dose group was significantly increased at 1 W after the 2nd immunization (*P* < *0.05*). The 0.5 g/mL and 2 g/mL dose groups significantly increased the content of IgG and IgG1 serum-specific antibodies (*P* < *0.05*), and the 0.5 g/mL dose group simultaneously promoted the secretion of serum IgG2a (*P* < *0.05*) 3 W after the 2nd immunization. There was no significant difference in antibody content among the other dose groups (*P* > *0.05*).

### mRNA expression levels of splenic-associated lymphocyte cytokines

**Figure 3:**
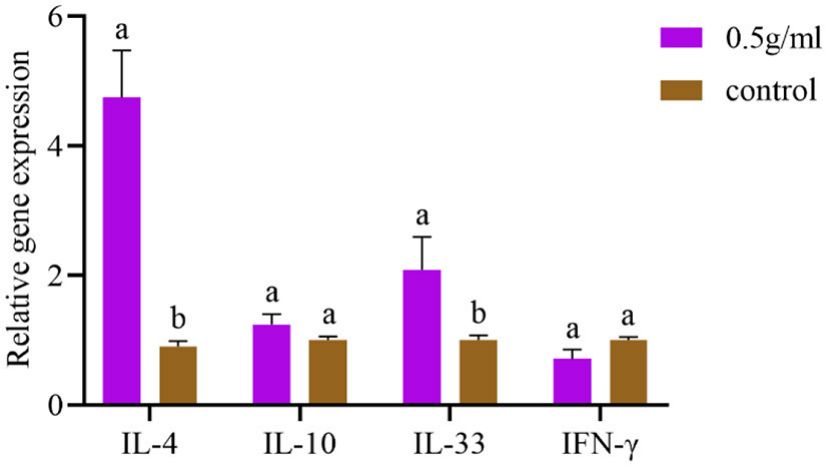
Effects of orally administered DBD on spleen cytokine mRNA expression in mice. ^a,b^Values in the same column with different superscript letters differ significantly at *P* < *0.05*.

As depicted in Figure 3 and compared with the control group, the mRNA expression levels of IL-4 and IL-33 in the spleen were significantly increased by oral DBD (*P* < *0.05*), and the mRNA expression levels of IL-10 and IFN-γ were not different from those of the control group (*P* > *0.05*).

### SIgA content of duodenal mucosal flushing fluid

**Figure 4:**
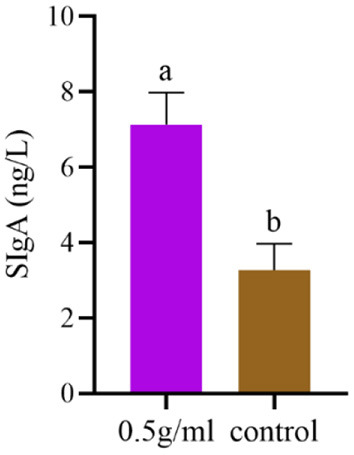
Effects of orally administered DBD on intestinal SIgA antibody content in the duodenum of mice. ^a,b^Values in the same column with different superscript letters differ significantly at *P* < *0.05*.

The effect of DBD on the SIgA content of duodenal mucosal flushing fluid is shown in Figure 4. The results suggest that 0.5 g/mL DBD significantly increased the intestinal SIgA antibody content (*P* < *0.05*).

### Relative mRNA expression of immune-related genes in the intestinal mucosa

**Figure 5:**
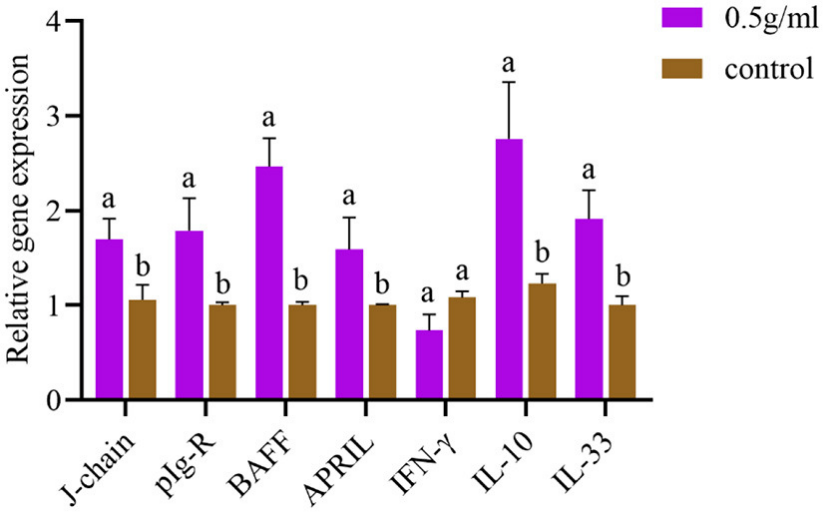
Effects of orally administered DBD on the mRNA expression of cytokines in the duodenum of mice. ^a,b^Values in the same column with different superscript letters differ significantly at *P* < *0.05*.

After oral administration of DBD, the mRNA expression levels of J-chain, pIgR, BAFF, APRIL, IL-10, and IL-33 in intestinal tissues were significantly increased compared with those in the control group (*P* < *0.05*). Additionally, the expression of IFN-γ was decreased, but there was no significant difference compared with the control group (*P* > *0.05*) (Figure 5).

## Discussion

Most modern pharmacological studies on the immunomodulatory effect are conducted by regulating non-specific immunity or evaluating the therapeutic effect of pathological models, while there are few studies on specific immunity (18). Therefore, this trial explored the effects of gastric infusion DBD on specific immunity. The organ index is the ratio of an organ to its weight in a test animal, which can reflect the strength of its function to a certain extent. The spleen, thymus, and liver are the most important immune organs in mammals, and the quality of animal immune organs is increased by their proliferation caused by cell growth and division. Therefore, the weight is proportional to the immune function of the body (19, 20). In this study, it was found that the organ index of mice after gavage of different doses of DBD was not significantly different from that of the control group, but the 0.5 g/ml and 2 g/ml dose groups were superior to the control group. The humoral immune response of the body mainly includes IgG, IgM, IgA, IgD and IgE. And IgG is the main effector molecule of humoral immune response. The level of IgG antibody in serum can be used as an indicator of the success of vaccination. According to the differences in the antigenicity of the γ chain in IgG molecules, IgG in mice has four isotypes of antibodies, namely, IgG1, IgG2a, IgG2b, and IgG3. The results of this test showed that the oral administration of DBD could improve the antibody level of FMDV antigen, which was consistent with the results of Sun et al. (21).

When FMDV infects animals, both humoral and cellular immune functions resist the pathogen. This study found that DBD can increase the specific antibody level and specific antibody of the mouse class level, which suggests that after lavage DBD, the change in the antibodies may be related to the change in immune cells and immune active substances in the body. During the body's cellular immune response, immune cells affect the production of antibodies by secreting cytokines, which also inhibit the proliferation of related cells (22). Although the mechanism of antibody generation is extremely complex, numerous studies have shown that specific cytokines can promote certain types of antibody subclasses switching and further to inhibit other antibody subclasses (23). CD4+ cells are divided into Th1 and Th2 subsets. Th1 subsets mainly express IFN-γ and IL-2, which are involved in cellular immunity to endogenous cell infection, while Th2 subsets express IL-4 and IL-10, which participate in allergic reactions and humoral immunity against parasitic infection (24). IFN-γ is an important B cell type switching factor that can induce B cells to secrete antigen-specific IgG2a (25). IL-4 can induce antigen-dependent IgG2a and IgE production (26) and enhance IgG1-mediated humoral immunity and NK-cell killing (27). In this study, the mRNA expression of IL-4 in the spleen was significantly increased after oral administration of DBD, but IL-10 and IFN-γ were not significantly changed, which may be due to the balance regulation of Th1 and Th2 cytokines in mice by DBD. Thus, DBD affects the distribution of antibodies and the synergistic inhibitory effect of IL-4 on IFN-γ (28).

The intestinal mucosa is the body's first line of defense against infection, which can induce effective mucosal immunity and generate a systemic immune response. Many studies have confirmed that oral administration of Chinese herbal medicine can enhance intestinal mucosal immune function (29, 30). As an important effector factor of mucosal immune response, SIgA can block the adhesion of pathogens to the mucosa, neutralize toxins and other bioactive antigens, and prevent a variety of microorganisms and mucosal antigens from entering intestinal epithelial cells. It does not require complement to form immune complexes with viruses in the respiratory tract, digestive tract and other parts to be excreted. Therefore, the concentration of SIgA antibody is commonly used clinically to evaluate the mucosal immune effect. The concentration of the SIgA is commonly used to evaluate the mucosal immune effect (31, 32). Studies have examined the immune-enhancing effect of *atractylodes* polysaccharide (33) and found that after gavage of *atractylodes* polysaccharide, the serum-specific IgG antibody and intestinal total SIgA content of mice vaccinated with FMD vaccine were significantly increased, which was similar to the results of this experiment. This suggests that the enhancement of the serum IgG response by Chinese herbal medicine is related to the enhancement of intestinal mucosal immunity. The IgA monomer produced by IgA+ plasma cells and the 15 kDa disulfide bond (J-chain) are connected in the cytoplasm and constantly aggregate to form the polymeric immunoglobulin receptor (pIgA). pIgA and the 80 kDa polymeric immunoglobulin receptor (pIgR) are combined and translocated to the surface of epithelial cells to form SIgA. However, PIgR can delay the degradation of SIgA without affecting the affinity of the antigen (34). Intestinal SIgA secretion is mediated by T cell-dependent and non-T cell-dependent immune regulation. In the T cell-dependent pathway, the interaction between antigen-presenting cells and Th2 cells releases Th2 cytokines, which promote the proliferation of IgA+ B cells and their differentiation into IgA-secreting plasma cells. Studies have shown that the dependence of T cells on mouse intestinal SIgA is also important (35) because mature B cells express IgA only after class switch recombination (CSR). In the T cell-independent response, B cells can be activated by triggering toll-like receptors, B cell receptor (BCR) antigen compounds or activated by DC-presented natural antigens, and the activation of the B cell activating factor of the TNF family (BAFF) and proliferation inducing ligand (APRIL) are important factors for IgA type switching and recombination (36, 37). IL-10 promotes the proliferation and differentiation of B cells into IgA-secreting plasma cells and also acts as an anti-inflammatory cytokine to inhibit NKT cell-mediated colitis and protect intestinal mucosa by regulating the expression of CD1d in intestinal epithelial cells (38, 39). The above discussion is involved, which needs to be further improved and explored its mechanism by our laboratory.

## Conclusion

In conclusion, oral DBD can improve the immune function of mice immunized with the O-type FMD vaccine by improving serum-specific antibodies and the intestinal mucosal immune response, which could boost the immune system and improve the immune effect of the FMD vaccine.

## Data availability statement

The original contributions presented in the study are included in the article/supplementary material, further inquiries can be directed to the corresponding author/s.

## Ethics statement

The animal study was reviewed and approved by Laboratory Animal Ethical Review Committee, Southwest University, Chongqing, China. Written informed consent was obtained from the owners for the participation of their animals in this study.

## Author contributions

BZ and LC conceived and designed the experiments. BZ, SB, and HD performed the experiments and collected and analyzed the data. HH and FG wrote the article, while HH, FG, and LC revised the article. All authors read and approved the final manuscript.

## Funding

This research was supported by Chongqing Research Program of Basic and Frontier Technology (cstc2020jcy-jmsxmX0418) and Fundamental Research Funds for the Central Universities (SWU120002).

## Conflict of interest

The authors declare that the research was conducted in the absence of any commercial or financial relationships that could be construed as a potential conflict of interest.

## Publisher's note

All claims expressed in this article are solely those of the authors and do not necessarily represent those of their affiliated organizations, or those of the publisher, the editors and the reviewers. Any product that may be evaluated in this article, or claim that may be made by its manufacturer, is not guaranteed or endorsed by the publisher.
